# Potentially Hazardous Drugs in the Paediatric ICU: A Narrative Review on the Exemplary Cases of Propofol, Chloramphenicol, and Acetylsalicylic Acid

**DOI:** 10.3390/children13040579

**Published:** 2026-04-21

**Authors:** Laura Beckers, Joery Verbruggen, Vera Saldien, Jozef De Dooy, Eva van Zanten, Thomas Peros, Miranda Wiggelinkhuizen, Philippe G. Jorens

**Affiliations:** 1 Anaesthesiology, Antwerp University Hospital, 2650 Edegem, Antwerp, Belgium; vera.saldien@uza.be; 2 (Paediatric) Critical Care Medicine, Antwerp University Hospital, 2650 Edegem, Antwerp, Belgium; joery.verbruggen@uza.be (J.V.); jozef.dedooy@uza.be (J.D.D.); eva.vanzanten@uza.be (E.v.Z.); thomas.peros@uza.be (T.P.); miranda.wiggelinkhuizen@uza.be (M.W.); philippe.jorens@uza.be (P.G.J.); 3 LEMP Faculty of Medicine and Health Sciences, University of Antwerp, 2610 Wilrijk, Antwerp, Belgium

**Keywords:** paediatric, children, drugs, adverse effects, critical care, propofol infusion syndrome, Reye’s syndrome

## Abstract

**Highlights:**

**What are the main findings?**

- Propofol, chloramphenicol, and acetylsalicylic acid should be avoided in the paediatric ICU based on the altered pharmacokinetics and/or pharmacodynamics that may be encountered in the critically ill child.
- Children in the PICU are at risk of adverse effects due to immature organ function, altered body composition, and drug interactions, leading to toxic accumulation, mitochondrial dysfunction, and fatal outcomes.

**What are the implications of the main findings?**

- These findings underline the need for strict indication, dose adjustment, and intensive monitoring when using these drugs in paediatric ICU patients, especially those with underlying mitochondrial disorders or multi-organ failure.
- The results support the development of guidelines and clinician education on safe alternatives and appropriate use to prevent unnecessary morbidity and mortality in paediatric intensive care.

**Abstract:**

**Background:** In the paediatric intensive care unit (PICU), certain drugs should be avoided or administered with strict precautions and close monitoring. This is due to their potential for toxicity or adverse effects or a lack of safety data, especially for critically ill children with organ failure. Additionally, practitioners must assess the unique pharmacokinetic and pharmacodynamic properties of drugs when treating critically ill children. In this narrative review, we highlight the risks, advantages, and disadvantages of three exemplary cases of drugs for paediatric patients hospitalised in the PICU: chloramphenicol, acetylsalicylic acid, and propofol. **Methods:** Apart from key papers on these drugs, a retrospective analysis of the English literature on chloramphenicol, acetylsalicylic acid (ASA), and propofol was performed on PubMed for papers from January 2014 to December 2025. **Results:** Chloramphenicol should be avoided in neonates due to the risk of grey baby syndrome. Acetylsalicylic acid (ASA) is contraindicated in children ≤18 years with suspected viral illness because of the risk of Reye’s syndrome, but remains essential for Kawasaki disease and post-cardiac surgery antiplatelet therapy. Propofol should be avoided when used for a longer period at high doses. With proper dosing and monitoring, propofol-related infusion syndrome (PRIS) is preventable, but high-risk patients should receive alternative treatment. **Conclusions:** This narrative review highlights the significant risks associated with the use of chloramphenicol, ASA, and propofol in paediatric intensive care settings. Their potential for life-threatening and severe adverse reactions emphasises the need for cautious and informed use. Clinicians must carefully consider the risks and benefits of these drugs. To minimise adverse events, strict monitoring, dose adjustments, and the use of safer alternatives are essential. However, it appears that their use in well-defined circumstances in acute illness in children is still warranted. The findings of this narrative review underscore the need for further research to focus on identifying high-risk biomarkers, genetic predispositions, and safer alternatives to improve evidence-based guidelines and reduce morbidity and mortality in paediatric intensive care.

## 1. Introduction

Paediatric patients have a unique susceptibility to adverse drug reactions. Some drugs may require extra caution when used in children due to their underdeveloped metabolic pathways or tissue systems. Conversely, other drugs may pose less risk, such as drugs with enhanced renal clearance in young, healthy kidneys. Historically and again in 2025, McPherson et al. identified 39 drugs and/or drug classes and 10 excipients that are likely unsuitable for use with all paediatric patients or a subgroup of them, which they entitled the KIDs List [1]. Especially in the intensive care setting, drugs must be selected with precision due to an increased risk of toxicity because of altered pharmacokinetics and dysfunctional organ systems.

In this narrative review, we discuss the adverse reactions observed with chloramphenicol, ASA, and propofol as exemplary cases. Why? Each of these drugs has led to severe, sometimes fatal, adverse reactions when used for critically ill children or infants. In addition, their inappropriate use in children outside the intensive care unit (ICU) has led to admission to the ICU due to sometimes even lethal severe adverse effects. They also represent overlapping pathways of toxicity (e.g., mitochondrial dysfunction, impaired drug metabolism, and dose-dependent organ toxicity) that are relevant in the PICU population. Furthermore, their use has evolved significantly throughout the years due to improved understanding of their risks, making them ideal for discussing medication safety, monitoring strategies, and the need for evidence-based alternatives.

This narrative review synthesises the key literature on these three drugs to provide clinicians with a comprehensive overview and highlight the need for strict indication, dose adjustment, and intensive monitoring when using these drugs. The results support the development of guidelines and clinician education on safe alternatives and appropriate use to prevent unnecessary morbidity and mortality in paediatric intensive care.

## 2. Methodology

Apart from historical key papers on these drugs, we performed an electronic search of the English-speaking literature in PubMed on chloramphenicol, ASA, and propofol for papers from January 2014 to December 2025.

The following search terms were used:

▪ For chloramphenicol: ‘chloramphenicol’, ‘infants’, ‘toxicity’, ‘grey baby syndrome’, ‘adverse drug reaction’, and ‘paediatric ICU’.
▪ For ASA: ‘acetylsalicylic acid’, ‘aspirin’, ‘ASA’, ‘paediatric ICU’, ‘critical care’, ‘ICU’, ‘Reye’, ‘children’, and ‘infants’.
▪ For propofol: ‘propofol infusion syndrome’, ‘paediatric ICU’, ‘infants’, ‘critical care’, and ‘children’.

The search strategy was as follows:

▪ For chloramphenicol: ‘chloramphenicol’ AND ‘infants’; ‘chloramphenicol’ AND ‘toxicity’ AND ‘infants’; ‘chloramphenicol’ AND ‘grey baby syndrome’; ‘chloramphenicol’ AND ‘adverse drug reaction’; ‘chloramphenicol’ AND ‘paediatric ICU’.
▪ For ASA: ‘acetylsalicylic acid’ AND ‘paediatric ICU’ AND ‘Reye’; ‘aspirin’ AND ‘paediatric ICU’ AND ‘Reye’; ‘paediatric ICU’ AND ‘Reye’; ‘Reye’ AND ‘infants’ AND ‘ICU’; ‘Reye’ AND ‘infants’ AND ‘critical care’; ‘Reye’ AND ‘children’ AND ‘critical care’; ‘Reye’ AND ‘children’; ‘Reye’ AND ‘infants’; ‘ASA’ AND ‘Reye’.
▪ For propofol: ‘propofol infusion syndrome’ AND ‘paediatric ICU’; ‘propofol infusion syndrome’ AND ‘infants’ AND ‘critical care’; ‘propofol infusion syndrome’ AND ‘children’ AND ‘critical care’; ‘propofol infusion syndrome’ AND ‘children’; ‘propofol infusion syndrome’ AND ‘infants’.

We screened the titles and abstracts of all identified articles to determine their eligibility. In total, 99 papers were identified. Non-English papers were excluded, and duplicate articles were manually deleted. Also, some papers could not be retained for full-text review due to a lack of access to the full text. Finally, 64 papers were retrieved for full-text review. We ultimately checked the reference lists of the selected articles in order to identify other relevant studies. Eventually, 55 articles were selected for use in the final analysis. No meta-analysis was performed.

## 3. Results

### 3.1. Pharmacological Considerations in Children

The physiology and pharmacology of children clearly differ from those of adults. Moreover, during growth, children undergo significant maturational changes in organ function and body composition, which lead to further variations in every stage of pharmacokinetics compared to adults, including absorption, distribution, metabolism, and elimination.

*Absorption* is the process by which a drug enters the bloodstream. As children age, variations occur in absorptive surfaces, gastric pH, gastric emptying time, gastrointestinal transit, maturity of secretions, and the activity of both bile and pancreatic fluids, all of which may influence the rate of drug absorption [2,3]. Compared with children, adults have more efficient absorption due to larger gastrointestinal tracts and more protein transporters [4].

In the drug *distribution* process, the drug is transported from the bloodstream to the target tissue. Drug distribution varies according to the blood flow, plasma protein binding of the drug, and body composition.

Infants and children have lower concentrations of key plasma binding proteins compared to adults. As a result, reduced protein binding increases the free, active fraction of highly protein-bound drugs. This increases the risk of toxicity or altered efficacy, especially in critically ill paediatric patients [5].

Moreover, body composition changes with age, which affects how drugs distribute in the body [6]. For example, the proportion of total body water decreases as children age [5]. In term neonates, total body water approximates 70%, but this declines to 60% at the end of the first 1–2 years and to less than 50% in adolescents [5,7]. At the same time, body muscle and fat gradually increase [7]. These differences in body composition have an influence on optimal drug dosing [6]. Greater total body water results in lower plasma concentrations for hydrophilic (water-soluble) drugs when given by weight. For lipophilic (fat-soluble) drugs, the opposite occurs [5].

Drug *metabolism* is the process by which drugs are broken down into smaller components. The major site for this process is within the liver [6]. Drug metabolism occurs through two major pathways: phase I and phase II reactions.

▪ Phase I reactions (e.g., oxidation, reduction, hydroxylation) modify the structure of the drug. The cytochrome P450 enzymes play a key role in this phase.
▪ Phase II reactions (e.g., glucuronidation, sulfation, methylation, acetylation of glutathione conjugation) further transform the drug. The UDP-glucuronyltransferase enzymes are the most important in this phase. The goal of this reaction is to increase the water solubility of drug metabolites, making them easier for the body to excrete.

Children have lower hepatic enzyme activity, which leads to slower and insufficient drug metabolism [2,3]. This limitation, for example, is particularly notable with drugs like chloramphenicol, which is primarily deactivated in the liver by the enzyme UDP-glucuronyltransferase. Neonates and young children have limited levels of this enzyme, which results in toxic accumulation of the drug [8,9].

*Elimination* is the process that removes drug molecules from the body. This primarily occurs through renal clearance (via urine), though drugs can also be eliminated via the lungs or by biliary excretion. Renal clearance of a drug involves three key processes:

▪ glomerular filtration: reaches adult values by 3 to 5 months,
▪ tubular secretion, and
▪ tubular reabsorption [6].

Tubular secretion and reabsorption mature much more slowly and do not reach adult levels until later in childhood. This delayed maturation can dramatically reduce drug clearance, resulting in the accumulation of drugs in the body [6]. Additionally, hepatic excretion of drug metabolites via bile is slower in children due to reduced gastrointestinal transit [2,4].

As a result, simple weight-based doses derived from adult pharmacokinetic/pharmacodynamic studies are insufficient and pose a risk for adverse drug reactions or underdosing in this vulnerable population. Moreover, the impact of pharmacogenetics on pharmacokinetics and pharmacodynamics in neonates and infants is largely unknown [10].

### 3.2. Pharmacodynamic Differences Between Critically Ill and Healthy Children

Pharmacology in critically ill children, like those treated in a paediatric intensive care unit (PICU), differs significantly from that in a regular paediatric ward due to many internal and external factors [11]. Internal factors include the critical illness itself, which can lead to:

▪ impaired liver and kidney function,
▪ reduced cardiac output, and
▪ altered drug distribution (e.g., in children with burns [12]).

These conditions impact the pharmacokinetics and pharmacodynamics of medications and may require changes in medication regimens, as well as closer monitoring of drug levels. External factors include:

▪ renal replacement therapy,
▪ extracorporeal membrane oxygenation (ECMO), and
▪ hypothermia.

Furthermore, in the PICU, children are often taking multiple medications simultaneously, which increases the potential for drug interactions. Therefore, drug interactions and the effect of one medication on the metabolism of another must be carefully considered in this setting.

### 3.3. Chloramphenicol: Risks, Toxicity, and Clinical Considerations

Chloramphenicol was discovered in 1947 and is predominantly a bacteriostatic broad-spectrum antibiotic that was initially isolated from extracts of *Streptococcus venezuelae* [8]. It is effective against various Gram-positive and Gram-negative bacteria and can be effective in treating serious anaerobic infections [13]. Originally developed to treat typhoid fever, its use has decreased due to widespread resistance in *Salmonella typhi*. Historically, it was also used empirically for paediatric patients with petechial rash and fever, given its strong efficacy against rickettsial infections [14]. Chloramphenicol penetrates the blood–brain barrier, making it effective for the treatment of bacterial meningitis and brain abscesses, particularly for infections caused by *Haemophilus influenzae*, *Streptococcus pneumoniae*, or *Neissera meningitidis*, given its bactericidal effect against these pathogens [8,13].

#### 3.3.1. Pharmacokinetics and Metabolism

The inactive succinate ester prodrug is the preparation available for intravenous administration. The prodrug must be broken down by the process of hydrolysis to release active chloramphenicol before the drug can work [8]. The bioavailability of active chloramphenicol from parenterally administered chloramphenicol succinate is highly variable in paediatric patients with normal kidney function. This is due to the renal elimination of the unchanged prodrug before the body has a chance to hydrolyse it to active chloramphenicol. It is important to note that in patients with renal dysfunction, such as those in the PICU, the bioavailability of chloramphenicol succinate may be increased [15,16].

Active chloramphenicol is primarily metabolised in the liver and deactivated through glucuronidation, which produces a non-toxic water-soluble metabolite that can be excreted via urine. Approximately 85% of its clearance from the body is liver-dependent, which means that serum levels of the drug need to be monitored in patients with underlying liver disease. Neonates and young children have limited UDP–glucuronyltransferase enzyme activity, which is essential for glucuronidation, resulting in an inability to efficiently metabolise chloramphenicol [8,9]. Furthermore, a reduced excretion of chloramphenicol by the neonatal kidneys leads to elevated serum levels of the drug and toxic accumulation [14].

#### 3.3.2. Adverse Effects and Toxicity

Initially thought to have no significant toxicity, it was later found to have serious side effects. In 1950, Rich et al. reported a case of aplastic anaemia linked to chloramphenicol [17]. Later, Volini et al. described a reversible, dose-related bone marrow suppression associated with this drug [18].

Regular monitoring of haemoglobin levels, blood cell counts, and reticulocyte levels is necessary. Monitoring should be performed before starting therapy with this drug and then every 3 to 4 days. If bone marrow suppression develops, it may be reversed by discontinuation of the drug or reduction of the drug dosage [13]. Unlike bone marrow suppression, chloramphenicol-associated aplastic anaemia is unrelated to the dosage and cannot be prevented by monitoring of blood cell counts as the irreversible stage of this disease occurs weeks to months after completion of therapy. Historically, this adverse event came with a high mortality rate of around 50% [8,13,19].

The first case report of a potentially fatal adverse reaction to chloramphenicol was 12 years after its discovery; the reaction is referred to as ‘grey baby syndrome’, which was discovered in neonates who had received high-dose chloramphenicol therapy. The syndrome typically presents 2–9 days after the initiation of chloramphenicol treatment and is characterised by abdominal distension, vomiting, respiratory distress, hypothermia, hypotension, progressive cyanosis, and ash-grey skin discolouration. In severe cases, grey baby syndrome can progress to cardiovascular collapse, severe metabolic acidosis, and eventually death if the drug is not immediately discontinued [14]. Immediate, aggressive supportive care is essential to stabilise the infant. Exchange transfusion should be considered as a treatment option, although it carries risks, especially in patients with cardiovascular collapse. Overdosage of chloramphenicol has also been successfully treated with charcoal-column hemoperfusion [13].

#### 3.3.3. Prevention

Prevention of adverse effects relies on avoiding the use of chloramphenicol in neonates and infants whenever possible. If its use is absolutely necessary, it is essential to ensure careful dosing, clinical monitoring of the patient, and therapeutic monitoring of drug serum levels every 48 h. The occurrence of grey baby syndrome is generally associated with serum levels exceeding 50 mg/L, so levels should generally be maintained between 15 and 25 mg/L [8,19,20].

#### 3.3.4. Conclusion and Clinical Implications

In conclusion, chloramphenicol was historically used for bacterial central nervous system infections and other severe infections. Though, its role in modern paediatric intensive care has significantly diminished due to the risk of potentially fatal adverse reactions. Grey baby syndrome can reach mortality rates of up to 40%. Fatal cases consistently show serum chloramphenicol levels exceeding 50 mg/L [9]. The availability of alternative antibiotics has further reduced its use in contemporary practice. Recent data continue to confirm the risks of chloramphenicol in paediatric populations. A 2022 systematic review by Eliakim-Raz et al. verified that neonates and infants remain at the highest risk for grey baby syndrome, particularly when doses exceed 25 mg/L [21]. Consequently, it can no longer be recommended as a first-line antibiotic in most PICUs [14,21]. Chloramphenicol remains particularly useful for specific cases where alternatives are limited or unavailable (e.g., in resource-limited settings or regions with high resistance to antibiotics) [14,21]. Healthcare workers should be aware of the risks of using chloramphenicol. When used, strict clinical and laboratory monitoring together with careful dosing and therapeutic drug monitoring is mandatory to mitigate risks [21].

Contemporary safety protocols for the use of chloramphenicol should include:

▪ Therapeutic drug monitoring to maintain serum levels between 15–25 mg/L and avoid toxicity (>50 mg/L) [19].
▪ Regular monitoring of haemoglobin levels, blood cell counts, and reticulocytes to detect early signs of bone marrow suppression.
▪ Immediate discontinuation if symptoms of grey baby syndrome (e.g., abdominal distension, vomiting, respiratory distress, hypothermia, hypotension, progressive cyanosis, and ash-grey skin discolouration) or bone marrow suppression emerge.
▪ Alternatives such as ceftriaxone or cefotaxime are now preferred for empirical treatment of meningitis in most cases [21].

### 3.4. Acetylsalicylic Acid: Risks of Reye’s Syndrome and Clinical Indications

Acetylsalicylic acid or aspirin (ASA) is one of the most widely used medications in adults and is known for its analgesic, antipyretic, anti-inflammatory, and antiplatelet effects. However, its use as an antipyretic is generally contraindicated in children because of its association with Reye’s syndrome, a rare but life-threatening condition that predominantly affects the liver and brain [3,22]. Even when the exact aetiology remains unknown, some epidemiologic studies have shown an association with the devastating Reye’s syndrome and ingestion of aspirin during viral illness, regardless of its dose [23,24,25,26]. Hall et al. found that over 80% of children diagnosed with Reye’s syndrome had taken aspirin within 3 weeks of onset [27]. The diagnosis of Reye’s syndrome is based on clinical signs, laboratory tests, liver biopsy findings, and the exclusion of other causes of acute encephalopathy and liver failure.

The primary viral pathogens associated with Reye’s syndrome are influenza A and B, as well as varicella zoster virus. There have also been links with other more rarely associated viruses, such as coxsackieviruses, parainfluenza viruses, Epstein–Barr virus, cytomegalovirus, and adenovirus. Additionally, the syndrome has been associated with infection by certain bacterial pathogens, including *Chlamydia*, *Bordetella pertussis*, *Mycoplasma*, and *Shigella* [22,28]. Most cases of Reye’s syndrome are seen in children in the age range of 4–12 years and almost exclusively occur below 15 years of age [29].

The incidence of Reye’s syndrome shows a pronounced seasonal pattern. Earlier US surveillance data (1980–1997) showed a peak in cases during winter months, particularly in children under 5 years of age. This seasonal trend reflects the prevalence of viral infections such as influenza, underscoring the epidemiological link between ASA use during viral illness and the onset of Reye’s syndrome [30]. This finding led to the introduction of public health policies discouraging ASA use in children with viral infections. As the use of ASA decreased from the 1980s onward, a significant decline in cases of Reye’s syndrome was observed in all countries with active surveillance (UK, US, and Ireland) [22,28,31].

#### 3.4.1. Pathophysiology

Given the high occurrence of these infections among children, only a small number actually develop Reye’s syndrome. Therefore, some researchers speculate that children who develop Reye’s syndrome may have an underlying genetic susceptibility to the condition [32]. The pathophysiology of aspirin-induced Reye’s syndrome, although the exact mechanism remains unknown, centres on mitochondrial dysfunction. The drug’s primary site of action is the mitochondrial trifunctional enzyme (MTE), specifically targeting long-chain hydroxyacyl-CoA dehydrogenase (LCHAD), a key component of the beta-oxidation system. Aspirin and its active metabolite, salicylate, inhibit LCHAD, impairing mitochondrial beta-oxidation of medium and long-chain fatty acids. This inhibition disrupts mitochondrial function, leading to energy failure in hepatocytes. This results in accumulation of fatty acids (microvesicular steatosis), hyperammonemia, and subsequent diffuse cerebral oedema and increased intracranial pressure, which explains the neurological symptoms seen in the vast majority of children with this syndrome [22,33,34].

Paediatric patients are more susceptible due to several factors. Children, especially those with underlying or unrecognised inborn errors of fatty acid metabolism, have less mature metabolic pathways, lower concentrations of hepatic mitochondrial enzymes, and limited capacity to compensate for mitochondrial dysfunction, making them more vulnerable to mitochondrial toxins such as salicylates [33]. Moreover, the syndrome is most often triggered in the context of viral stressors, which can further compromise mitochondrial function and increase susceptibility to aspirin toxicity. As previously described, epidemiologic data show a decline in Reye’s syndrome incidence after public health warnings against aspirin use in children, supporting the causal role of aspirin and the particular vulnerability of the paediatric population [22,28,31,33].

#### 3.4.2. Clinical Presentation

The disease is typically biphasic, meaning that it usually develops during a viral infection, followed a few days later by persistent vomiting, changes in the mental state, and various grades of decreased consciousness [31,35]. The Centres for Disease Control and Prevention (CDC) describe this clinical progression using five grades, with symptoms escalating rapidly within hours to days following the initial infection, as shown in Table 1 [22,28,31,36].

There is no sensitive or specific test for Reye’s syndrome [22,29]. The differential diagnosis is complicated and includes encephalitis to meningitis, various intoxication syndromes, viral hepatitis, chronic liver diseases during acute exacerbation, poisoning with hepatotoxic substances, and inborn errors of metabolism. The CDC has defined three mandatory criteria for suspicion of a diagnosis of Reye’s syndrome [22,28,31,36]:

- Acute, non-inflammatory (and metabolic) encephalopathy that is documented clinically with alterations in the level of consciousness and, if available, a record of cerebrospinal fluid containing a leukocyte count of 8 cells/mm^3^ or fewer, or by a histological specimen demonstrating cerebral oedema without perivascular or meningeal inflammation
- Acute liver failure (characterised by fatty degeneration of the liver) documented by either a liver biopsy or an autopsy considered to be diagnostic of Reye’s syndrome, or an increase of three-fold or greater in the levels of serum transaminases and/or ammonia
- No other reasonable explanation for the cerebral and hepatic abnormalities.

#### 3.4.3. Treatment and Prognosis

Early recognition of Reye’s syndrome and prompt referral to an intensive care unit are crucial. Studies have shown that a delay in transfer to the PICU has a significant negative impact on the patient’s outcome [37]. Treatment remains supportive and involves close clinical monitoring, which is ideally performed in an intensive care setting. Effective management of raised intracranial pressure is essential for survival. In about 30–40% of cases, death occurs from brainstem dysfunction [36]. In grade I disease, the recovery is complete. If a child has already advanced to coma grade IV or V, survival is unlikely [22,37].

The overall mortality of Reye’s syndrome is as high as 85%; thus, prevention remains the primary focus [29,36]. Public awareness concerning the possible severe consequences of aspirin use during viral infections is essential. It has led to the abandonment of the use of aspirin as an antipyretic.

#### 3.4.4. Conclusion and Clinical Implications

As a summary, ASA is strictly contraindicated in children ≤18 years with suspected or confirmed viral illness due to the risk of Reye’s syndrome. Nevertheless, there are specific medical conditions where aspirin has its value in the PICU:

- After congenital heart surgery with stents, shunts, or valves, low-dose ASA (3–5 mg/kg/day) provides an antiplatelet effect that reduces the risk of thrombosis [38].
- In cases of Kawasaki disease, high-dose ASA (80–100 mg/kg/day) remains a mainstay of treatment. The goal in the treatment of this disease is to rapidly reduce systemic inflammation, followed by low-dose ASA (3–5 mg/kg/day) for antiplatelet effects [38].
- For patients with multisystem inflammatory syndrome (MIS-C), aspirin has a key role in therapeutic antiplatelet and anti-inflammatory effects, often in combination with corticosteroids. Some studies suggest low-dose ASA (3–5 mg/kg/day) to be given to patients with MIS-C, others recommend an increased ASA dose of 80–100 mg/kg/day in case of raised inflammatory markers (ferritin > 700 mg/mL or CRP > 30 g/dL) or cardiac involvement [39].
- Furthermore, it plays a significant role in the treatment of acute pericarditis (medium-dose ASA 30–50 mg/kg/day) in children by reducing inflammation and relieving symptoms such as chest pain. In some cases, aspirin is efficacious in combination with colchicine in the treatment of the first episode of acute pericarditis, as well as in the prevention of recurrences. Colchicine has been shown to reduce the recurrence rate when added to aspirin therapy [40].

Given the risk of Reye’s syndrome, the use of ASA demands strict biochemical and clinical monitoring. Liver function tests should be performed at baseline and monitored periodically to detect early signs of hepatic dysfunction. Clinicians must also remain vigilant for symptoms of Reye’s syndrome, including persistent vomiting, altered mental status, or hepatomegaly. Immediate discontinuation of ASA is mandatory if any of these signs emerge, particularly in the context of a recent or ongoing viral illness. To mitigate risks, alternatives should always be considered: paracetamol (acetaminophen) or ibuprofen can replace ASA for antipyresis and analgesia, while corticosteroids and intravenous immune globulin are preferred for anti-inflammatory indications. For anticoagulant purposes, low-molecular-weight heparin (LMWH) may serve as a safer option when ASA is contraindicated.

Public health policies and clinician education should continue to emphasise the risk of Reye’s syndrome and the importance of avoiding ASA during viral illness.

### 3.5. Propofol-Related Infusion Syndrome: Pathophysiology, Risks, and Clinical Management

Propofol is one of the most frequently used intravenous hypnotic anaesthetic drugs because of its favourable pharmacokinetic characteristics. It helps maintain procedural sedation and hypnosis during general anaesthesia for surgery. Additionally, propofol is a desirable choice for sedating mechanically ventilated patients in adult intensive care units. Its rapid onset of action and short half-life enable faster awakening after discontinuing the infusion. Moreover, the dosage of propofol can be easily adjusted for critically ill patients to ensure an optimal degree of sedation [41]. However, the use of propofol comes with risks and side effects. Propofol-related infusion syndrome (PRIS) is a rare but usually fatal complication associated with the prolonged high-dose administration of propofol [42]. It was first seen in the paediatric population and later in adults as well [41].

#### 3.5.1. Definition and Clinical Features of PRIS

**Figure 1:**
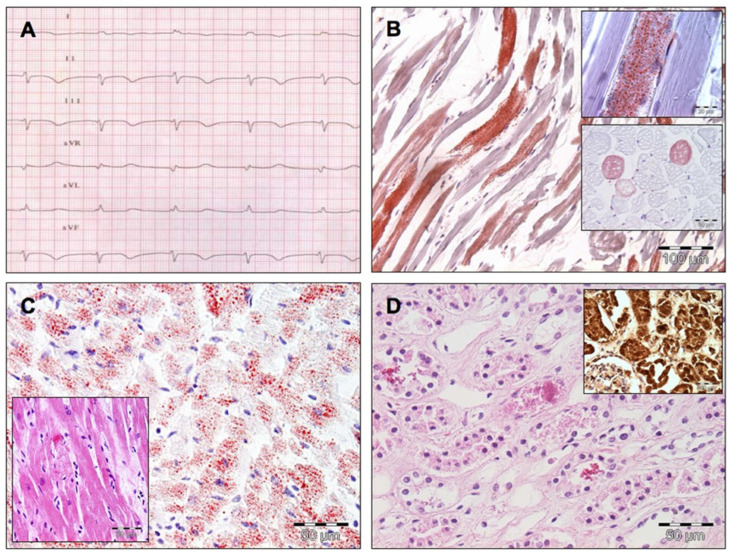
A case of massive PRIS. (**A**) Electrocardiogram depicting a sudden nodal bradyarrhythmia. Note the coved-type ST-segment elevation in the right precordial leads. (**B**) Post-mortem examination showed an accumulation of fat droplets and acute vacuolar degeneration and myocytolysis of skeletal muscle (oil red O staining stains the accumulated fat red). (**C**) Not only skeletal but especially cardiac muscle showed swelling, vacuolar degeneration, and myocytolysis. Moreover, the oil red O staining again shows widespread accumulation of fat (inset: haematoxylin eosin staining illustrates the subendocardial acute necrotic reaction with loss of striation). The lack of a significant inflammatory infiltrate illustrates the acuity of the process. (**D**) Acute tubular necrosis and massive reddish brown, myoglobin-immunoreactive pigment casts in the renal tubular lumina, a hallmark of myoglobinuria because of the massive rhabdomyolysis. (Reprinted with permission from Jorens et al. [42]. Copyright 2009, Elsevier.)

In 1998, Bray formulated the term PRIS to describe this clinical state. The clinical features include acute refractory bradycardia (as seen in Figure 1) leading to asystole, combined with one or more of the following conditions:

▪ clinically enlarged liver or fatty infiltration at autopsy,
▪ marked hyperlipidaemia,
▪ metabolic acidosis with base excess less than −10 mmol/L, or
▪ signs of skeletal muscle involvement assessed by myoglobinuria or rhabdomyolysis.

In addition, lactic acidosis, arrhythmia, renal, cardiac, and circulatory failure can occur [43].

PRIS has a broad clinical presentation, but cardiovascular and metabolic symptoms are most common in both adults and children. The clinical features of PRIS can differ somewhat between adults and children, although there is significant overlap. Lipidaemia, fever, and hepatomegaly occur more frequently in children than in adults, whereas adults are more likely to experience rhabdomyolysis and hyperkalaemia [44]. Mortality from PRIS is independently associated with fever and hepatomegaly in children, electrocardiogram changes, hypotension, hyperkalaemia, traumatic brain injury, and an elevated mean propofol infusion rate, which are linked with a poor prognosis, in children and adults too [44].

#### 3.5.2. Pathophysiology of PRIS

**Figure 2:**
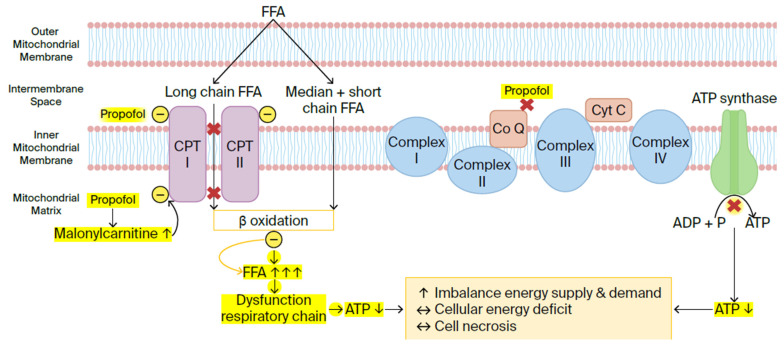
Pathophysiological mechanism of PRIS (the figure was made by the authors of this article, with adaptations from Vasile et al. [45]).

The exact mechanism responsible for PRIS remains unclear, but it appears that the primary pathophysiology behind PRIS involves mitochondrial dysfunction with disruption of the mitochondrial respiratory chain (Figure 2).

The mitochondrial respiratory chain consists of four enzyme complexes (complexes I–IV), as well as two mobile electron carriers: ubiquinone (coenzyme Q) and cytochrome C. These molecules are found in the inner mitochondrial membrane, where they form a continuous reaction system. Generally, the movement of electrons in the mitochondrial respiratory chain causes proton translocation and accumulation in the inner mitochondrial membrane, which results in an electromechanical gradient that promotes the production of adenosine triphosphate (ATP). Cells use ATP as their main energy source, which makes it essential for cellular function. The disruption of the mitochondrial respiratory chain by propofol reduces the mitochondria’s ability to produce ATP. This can lead to an imbalance between energy demand and utilisation, causing cellular hypoxia and, eventually, metabolic acidosis.

Based on in vitro research and animal studies, Kam and Cardone suggested in their review that these effects occur either through the inhibition of coenzyme Q, blocking electron transport from complex II to complex III, or through the disruption of fatty acid oxidation by inhibiting key transporters, carnitine palmitoyl transferase I and II (CPTI/II) [46]. Fatty acid transporters help move long-chain fatty acids into the mitochondrial matrix, whereas short- and medium-chain fatty acids can freely diffuse across the mitochondrial membrane. Inside the mitochondrial matrix, fatty acids undergo metabolisation through beta-oxidation. Inhibition of fatty acid transporters causes fatty acids to accumulate in the mitochondria, which leads to dysfunction of the respiratory chain, resulting in a cascade of reduced ATP production, cellular hypoxia, and metabolic acidosis [44,46,47]. Vanlander et al. demonstrated in their study on rats that propofol inhibits coenzyme Q, a finding similar to that of Kam and Cardone. They hypothesised that high-dose, prolonged propofol use causes its incorporation into the inner mitochondrial membrane. There, it interrupts electron flow in the respiratory chain, primarily at the coenzyme Q site [48]. The excess propofol itself, not its metabolites, is directly responsible for the mitochondrial dysfunction seen in PRIS [48,49]. Furthermore, Wolf et al. reported in a patient case report that propofol causes an increase in serum malonyl carnitine, which is an inhibitor of CPT I [47].

Most insights concerning the pathophysiology of PRIS are supported by preclinical studies, and clinical confirmation of these mechanisms in humans remains limited. Nevertheless, we can conclude that propofol seems to cause multiple metabolic changes in the mitochondria. These changes disrupt the balance between energy supply and demand, resulting in a cellular energy deficit. Ultimately, this imbalance may potentially lead to cardiac and peripheral muscle cell death and necrosis.

Paediatric patients may be more susceptible to PRIS due to several factors. First, children require higher blood concentrations of propofol for anaesthesia, increasing their exposure to potential mitochondrial toxicity. Secondly, they have less mature mitochondrial respiratory chain function and lower reserves of coenzyme Q, making them more vulnerable to energy failure when exposed to mitochondrial toxins like propofol [49,50]. Additionally, paediatric patients may have a higher prevalence of undiagnosed or subclinical mitochondrial diseases, further elevating their risk [46,50]. Moreover, children exhibit lower rates of propofol metabolism and excretion compared to adults, leading to prolonged drug exposure.

In critically ill patients, where lipids serve as the primary energy source instead of carbohydrates, excess lipolysis of adipose tissue occurs [45]. This process generates an excess of free fatty acids that cannot undergo adequate beta-oxidation, contributing to the clinical pathology of PRIS [41] (Figure 2). Early adequate carbohydrate intake may help prevent PRIS by preventing the switch to fat metabolism [47]. The larger carbohydrate stores in adults could explain their lower risk of developing this syndrome.

#### 3.5.3. Clinical Presentation and Risk Factors for PRIS

PRIS is primarily characterised by severe cardiovascular dysfunction along with high-anion-gap metabolic acidosis due to lactic acidosis and renal failure as a result of cellular hypoxia. Depletion of ATP and elevated free fatty acids severely compromise the cardiomyocyte function. The direct toxic and pro-arrhythmic effects of free fatty acids contribute to ventricular arrhythmias [41,42,46]. Post-mortem investigation shows myocytolysis in skeletal and cardiac muscle (see Figure 1) and widespread fat accumulation in the myocardium. This illustrates the underlying pathophysiology of impaired free-fatty-acid utilisation [42]. Furthermore, propofol antagonises β-adrenoceptor binding and acts directly on calcium-channel proteins, resulting in diminished cardiac contractility [45,51]. These mechanisms contribute to catecholamine resistance and the need to escalate inotropic support seen in critically ill patients.

Hepatic dysfunction is common in PRIS and may be caused by multiple mechanisms. Bray suggested that hepatic congestion from cardiac failure contributes to these findings [43]. Furthermore, the high levels of lipid deposition from propofol and free fatty acids are further responsible for the enlargement of the liver and impairment of its function [43]. Other hallmark manifestations include rhabdomyolysis, hyperkalaemia, lipidaemia, and acute renal failure [41].

Multiple risk factors have been identified in the development of PRIS, highlighting the importance of careful patient selection in the PICU. According to Singh et al. and Hemphill et al., the significant risk factors that predispose one to PRIS are:

▪ poor oxygen saturation,
▪ sepsis,
▪ neurological injury,
▪ ongoing critical illness,
▪ young age (significantly below 3 years),
▪ catecholamine and corticosteroid infusion,
▪ obesity,
▪ depleted carbohydrate stores in the body,
▪ increased serum lipids, and
▪ inborn errors of metabolism [41,44].

Based on the available medical literature, Finsterer and Frank further emphasise that patients with a mitochondrial disorder are likely to have a higher risk of developing PRIS [50]. In patients with pre-existing mitochondrial disorders, propofol may further impair mitochondrial function. Patients with a mitochondrial disorder, even adults, should not receive propofol at high doses for a prolonged period of time [52]. Short-term application of propofol, even at high dosages, should be safe even in cases of mitochondrial disorder [50].

**Figure 3:**
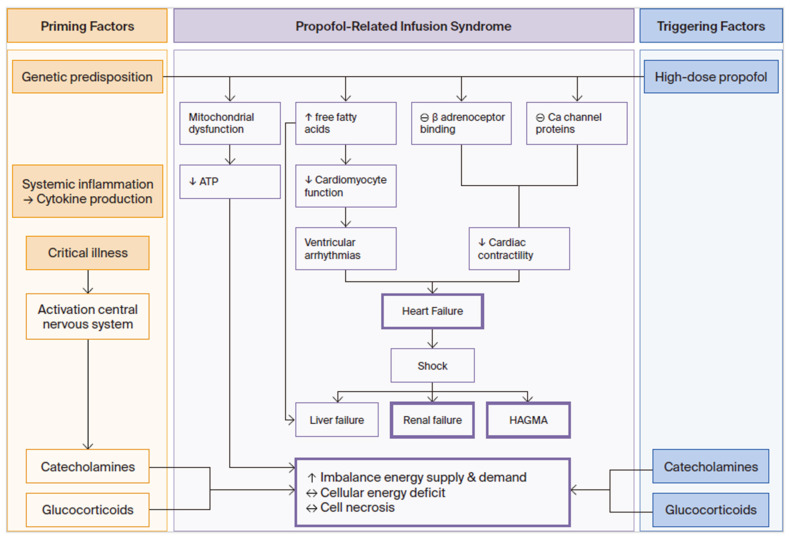
Factors that predispose to the propofol infusion syndrome. (The figure was made by the authors of this article, with adaptations from Kam and Cardone [46]).

Vasile et al. and Kam et al. proposed ‘priming factors’ and ‘triggering factors’ that contribute to PRIS [45,46] (Figure 3). Priming factors for cardiac and peripheral muscle dysfunction include activation of the central nervous system in critical illness with production of catecholamines and glucocorticoids, as well as systemic inflammation with cytokine production. Triggering factors include high-dose propofol, as well as supportive treatments with exogenous catecholamines and corticosteroids. All of these can exacerbate the imbalance between energy demand and utilisation in children [45,46]. Hemphill et al. suggested that the cumulative dose of propofol is the most important risk factor in the aetiology of PRIS, either through high infusion rates (>4 mg/kg/h), prolonged duration of infusion (>48 h), or both. However, cases have occurred after infusions with low doses and short duration, highlighting the need for clinical vigilance even outside the traditionally high-risk scenarios [44]. Wolf et al. suggested that a carbohydrate intake of 6–8 mg/kg/min could suppress fat metabolism in critically ill children and thus prevent PRIS, but this is not implemented in daily practice [47].

#### 3.5.4. Conclusion and Clinical Implications

On the other hand, the debate remains open. Recent studies have shown conflicting evidence regarding its incidence and preventability. For instance, Takeshita et al. reported an extremely low occurrence of PRIS in a large retrospective analysis of a nationwide hospital-based database in Japan, even when propofol was used as part of a total intravenous anaesthesia technique [53]. Moreover, in another retrospective study, Moas et al. found no cases of PRIS in critically ill paediatric patients receiving propofol infusions at doses higher and durations greater than the described safety data in the PICU, as long as strict monitoring protocols (e.g., lactate levels, biochemical characteristics, and ECG changes for PRIS) were followed [54]. Despite these reassuring findings, the overall incidence of PRIS in the ICU remains low but significant, ranging from 1.1% to 4.4%. However, once PRIS develops, it is associated with high mortality rates of 46% to 64%, underscoring the need for vigilance and proactive prevention [53].

These findings suggest that PRIS may be preventable with appropriate safety measures, rather than an inevitable consequence of propofol use. However, other studies, such as those of Hemphill et al., emphasise that high cumulative doses (> 4 mg/kg/h for > 48 h) remain the most important risk factor, particularly in patients with underlying mitochondrial disorders or critical illness [44].

Clinical implications:

▪ Propofol should not be categorically avoided in the PICU but should be used with strict adherence to dosing limits and continuous clinical and metabolic monitoring. It is recommended to avoid propofol infusions at a rate greater than 4 mg/kg/h for longer than 48 h [44,46]. The use of a more concentrated propofol solution can help to reduce the lipid load [46].
▪ Additionally, propofol should be used in combination with other sedative agents. In cases where alternatives (e.g., dexmedetomidine, midazolam) are unavailable, short-term and low-dose propofol may still be considered, provided that risk factors (e.g., sepsis, catecholamine use) are carefully assessed.

The successful management of PRIS relies on rapid recognition of its early signs and symptoms and timely interventions. In the event of any abnormality, the propofol infusion should be stopped immediately and an alternative sedation agent should be used. Several studies recommend early consideration of continuous renal replacement therapy in the management of PRIS [44]. Otherwise, treatment remains mainly supportive. Severe cardiovascular collapse can necessitate mechanical circulatory support, such as ventricular assist devices and extracorporeal membrane oxygenators [55].

Clinicians need to be informed about the condition and what its clinical features are. Maintaining a high level of awareness is essential for all patients receiving high-dose short-term or long-term infusions.

## 4. Discussion

This narrative review highlights the significant risks associated with the use of chloramphenicol, ASA, and propofol in paediatric intensive care settings. Our findings align closely with the 2025 KIDs List recommendations [1], emphasising the need for cautious and informed use when prescribing these drugs to critically ill children.

▪ Chloramphenicol should be avoided in neonates, as strongly recommended by the KIDs List (high-quality evidence), due to the risk of toxic accumulation and grey baby syndrome. In resource-limited settings where alternatives are unavailable, chloramphenicol can be used, but only if therapeutic drug monitoring (serum levels of 15–25 mg/L) and daily haematological monitoring are feasible to mitigate risks.
▪ ASA is contraindicated in children ≤ 18 years with suspected viral illness, reflecting the KIDs List’s weak recommendation (very low-quality evidence)**,** due to the risk of Reye’s syndrome. Its use should be restricted to certain well-defined and severe inflammatory disorders and for post-cardiac surgery antiplatelet therapy.
▪ Propofol, though safe for use during short anaesthetic procedures, should be avoided when used for a longer period at high doses (> 4 mg/kg/h and > 48h), as strongly recommended by the KIDs List (moderate-quality evidence). Recent data confirm that PRIS is preventable with proper dosing and monitoring, but high-risk patients (e.g., mitochondrial disorders) should receive alternative sedation (e.g., dexmedetomidine).

The risks associated with using these drugs are further increased by additional challenges in paediatric intensive care, such as polypharmacy-related interactions and inappropriate prescribing. Addressing these issues is essential to prevent avoidable harm.

Clinicians must carefully consider the risks and benefits of these drugs in paediatric patients. Strict monitoring, dose adjustments, and the use of safer alternatives are essential to minimise adverse events. Public health policies and clinician education should continue to emphasise the danger of ASA during viral illness.

Recommendations for practice:

▪ Monitoring: strict monitoring of serum drug levels (chloramphenicol), ECG, and metabolic parameters is crucial when using chloramphenicol or propofol.
▪ Dose adjustments: doses should be adapted to the patient’s age, weight, and organ function.
▪ Alternatives: safer alternatives, such as dexmedetomidine and midazolam for sedation and other antibiotics, i.e., ceftriaxone or cephalosporines for infections, should be considered where possible.

Future research is needed to identify biomarkers for high-risk patients, understand genetic predispositions, and evaluate the efficacy and safety of alternative treatments in paediatric intensive care settings. Developing evidence-based guidelines will be crucial to improving patient outcomes and minimising unnecessary morbidity and mortality.

## 5. Conclusions

Recognising potential lethality and severe adverse reactions is an essential first step to improving medication safety if chloramphenicol, ASA, or propofol is used in the PICU.

Based on experience with neonates, chloramphenicol is not a first-choice antibiotic, even for infants with septic shock. In resource-limited settings where safer alternatives are unavailable, its use may be justified, but only if rigorous therapeutic drug monitoring and clinical surveillance are feasible to mitigate the risk of grey baby syndrome.

Aspirin should be restricted to post-cardiac surgery antiplatelet therapy and certain well-defined and severe inflammatory disorders, although high doses may be used in these cases. This restriction reflects the delicate balance between its proven efficacy in conditions like Kawasaki disease and the risks of Reye’s syndrome during viral illness.

While propofol is safe for use during short anaesthetic procedures, it should not be used for a longer period at high doses in cases of central neurological disorders in children. Recent data confirm that PRIS is preventable with proper dosing and monitoring, but high-risk patients should receive alternative treatment.

Ultimately, the safe use of these drugs in the PICU relies on a dynamic, patient-centred approach. When using these drugs, you must consider several complex factors and challenges related to paediatric intensive care. These include the patient’s multimorbidity and possible genetic predispositions, unique pharmacokinetics and pharmacodynamics, and polypharmacy-related interactions, among others. A careful risk–benefit assessment together with daily clinical monitoring is crucial.

## Figures and Tables

**Table 1:** Clinical staging of Reye’s syndrome.

Grade	Symptoms
I	Lethargy, persistent and copious vomiting, and laboratory evidence of hepatic dysfunction
II	Profound lethargy, disorientation, hyperreflexia, and a positive Babinski sign
III	Obtundation, light coma, decorticate rigidity with preserved pupillary reaction
IV	Deep coma with decerebrate rigidity, seizures, absence of oculocephalic reflexes, and fixed pupils
V	Coma, loss of deep tendon reflexes, fixed and dilated pupils, decerebrate rigidity, respiratory arrest, and, eventually, death

## Data Availability

No new data were created or analysed in this study.

## Abbreviations

The following abbreviations are used in this manuscript:

PICU	Paediatric Intensive Care Unit
ASA	Acetylsalicylic Acid
PRIS	Propofol-Related Infusion Syndrome
ECMO	Extracorporeal Membrane Oxygenation
CDC	Centres for Disease Control and Prevention
MIS-C	Multisystem Inflammatory Syndrome in Children
LCHAD	Long-Chain Hydroxyacyl-CoA Dehydrogenase
MTE	Mitochondrial Trifunctional Enzyme
CPT	Carnitine Palmitoyl Transferase
ATP	Adenosine Triphosphate
CPT I/II	Carnitine Palmitoyl Transferase I/II
ICU	Intensive Care Unit
KIDs List	Key Potentially Inappropriate Drugs in Paediatrics
UDP	Uridine Diphosphate
LMWH	Low-Molecular-Weight Heparin
FFA	Free Fatty Acids
CoQ	Coenzyme Q
CytC	Cytochrome C
HAGMA	High Anion Gap Metabolic Acidosis
